# Selenium biofortification of soybean genotypes in a tropical soil *via* Se-enriched phosphate fertilizers

**DOI:** 10.3389/fpls.2022.988140

**Published:** 2022-09-14

**Authors:** Maila Adriely Silva, Gustavo Ferreira de Sousa, Ana Paula Branco Corguinha, Josimar Henrique de Lima Lessa, Guilherme Soares Dinali, Cynthia Oliveira, Guilherme Lopes, Douglas Amaral, Patrick Brown, Luiz Roberto Guimarães Guilherme

**Affiliations:** ^1^Soil Science Department, Federal University of Lavras, Lavras, Brazil; ^2^ICL South American, São Paulo, Brazil; ^3^University of California, Handord—Agriculture and Natural Resources, Hanford, CA, United States; ^4^Department of Plant Science, University of California, Davis, Davis, CA, United States

**Keywords:** biofortification, food security, cereal, nutritional quality, selenate

## Abstract

Soybean is a major crop in Brazil and is usually grown in oxidic soils that need high rates of phosphate (P) fertilizers. Soybean is also very suitable for biofortification with Se, since its grains have high protein contents and are widely consumed worldwide (directly or indirectly). Few studies have addressed Se application under field conditions for soybean biofortification, especially in tropical soils. Here, we evaluated agronomic and physiological responses resulting from different strategies for biofortifying soybean grains with Se by applying this element *via* soil, using both conventional and enhanced-efficiency P fertilizers as Se carriers. The experiment was carried out at the Uva Farm, in Capão Bonito (São Paulo), Brazil. The experimental design was a randomized block split-plot design, with four fertilizer sources—conventional monoammonium phosphate (C-MAP), conventional monoammonium phosphate + Se (C-MAP + Se), enhanced-efficiency monoammonium phosphate (E-MAP), and enhanced-efficiency monoammonium phosphate + Se (E-MAP + Se), and four soybean genotypes (M5917, 58I60 LANÇA, TMG7061, and NA5909). The selenium rate applied *via* C-MAP + Se and E-MAP + Se was 80 g ha^−1^. The application of the tested fertilizers was carried out at the sowing of the 2018/2019 cropping season, with their residual effect being also assessed in the 2019/2020 cropping season. Selenium application increased grain yield for the TMG7061 genotype. For all evaluated genotypes, Se content in grains increased in the 2018/2019 harvest with the application of Se *via* C-MAP + Se and E-MAP + Se. In general, the application of Se *via* C-MAP favored an increase in amino acid contents in grains and decreased lipid peroxidation. In summary, the application of Se-enriched P fertilizers *via* soil increased soybean grain yield, leading to better grain quality. No residual effects for biofortifying soybean grains were detected in a subsequent soybean cropping season.

## Introduction

Selenium (Se) is an essential element for humans and animals. It is a component of selenoaminoacids (e.g., selenocysteine), being necessary for the synthesis of more than 25 selenoproteins (Rayman, 2012; Oliver and Gregory, 2015). As a component of glutathione peroxidase, Se acts against oxidative stresses. In addition, Se also participates in thyroid metabolism and the immune system maintenance, reducing cancer and heart disease (Rayman, 2012; Avery and Hoffmann, 2018). It is estimated that about 1 billion people worldwide are Se deficient (Mora et al., 2015). Keshan and Kashin-Beck diseases are associated with Se deficiency in human organisms. Keshan is related to cardiomyopathy affecting children and young women and Keschin-Beck is related to osteoarthritis, promoting bone atrophy (Yao et al., 2011).

Selenium is not currently considered a plant nutrient though its beneficial effects on vegetables have been studied for over 70 years (Lyons et al., 2009; Feng et al., 2013). Several beneficial effects of this element for plants have been reported, such as improved rice growth (Boldrin et al., 2012), increased photosynthetic rate and wheat yield (Lara et al., 2019), reduced production of free radicals in lettuce (Ramos et al., 2011), increased protein content and total amino acids in soybean (Zhao et al., 2019), and reduced the damage caused by water stress in rice and common bean plants (Andrade et al., 2018; Ravello et al., 2021). For this reason, due to new trends in plant nutrient classification, Se and other beneficial elements (Na, Si, Al, Co, and I) may be considered plant nutrients in the future (Brown et al., 2021).

Selenium availability in soils depends on several factors, such as the Se source, soil mineralogy, redox condition, pH, and the presence of other anions (Lopes et al., 2017). Tropical soils are known for their high capacity to retain oxyanions—including selenite and selenate—with Se availability being decreased with increasing clay content. This is due to the high concentration of Fe/Al oxyhydroxides present in oxidic soils from tropical regions (Lopes et al., 2017; Araujo et al., 2018). Because of that, plants grown in soils with low Se concentration and availability show inadequate accumulation of this element in their edible parts (White and Broadley, 2009).

The adoption of biofortification practices is a suitable strategy to increase Se contents in food crops. Biofortification is a strategy that aims to increase the content of minerals and vitamins in crops *via* genetic (e.g., breeding) and/or agronomic (fertilization) practices (Cakmak, 2008; White and Broadley, 2009). Knowing the various constraints related to Se availability in Brazilian agroecosystems, the Brazilian Ministry of Agriculture, Livestock, and Supply approved a new legislation (normative N° 46/2016), which allowed the addition of Se in fertilizers marketed in Brazil (Brazil, 2016). A possible and relevant alternative to directly applying Se fertilizers in tropical agroecosystems could be its co-application *via* phosphate fertilizers, since the presence of competing anions, such as phosphate, reduces Se adsorption, increasing soil Se availability (Lessa et al., 2016; Mateus et al., 2021). Studies involving the biofortification of rice grown in tropical soils have reported the efficacy of the strategy of supplying Se to plants *via* its co-application with monoammonium phosphate—MAP (Lessa et al., 2020). Many P-fertilizer products are currently being used in oxidic soils with a technology to reduce phosphate retention (e.g., the so-called enhanced-efficiency products), it is thus relevant to determine if such technologies could improve Se use efficiency when selenium is soil-applied using enhanced-efficiency MAP as a carrier.

Additional studies evaluating Se application *via* soil associated with sources of phosphate fertilizers are still required. To the best of our knowledge, there are few studies in tropical soils assessing Se application, mainly focusing on the co-application of Se with phosphate fertilizers. Soybean is an interesting agricultural crop for biofortification with Se due to the large number of products generated from soybean grains, the high concentration of proteins, and the geographic distribution of soybean production. The present study aimed to evaluate the effectiveness of applying Se in association with phosphate fertilizers for soybean biofortification and its residual effect in the succeeding cropping season in tropical soils.

## Materials and methods

### Experimental area and treatments

**Figure 1:**
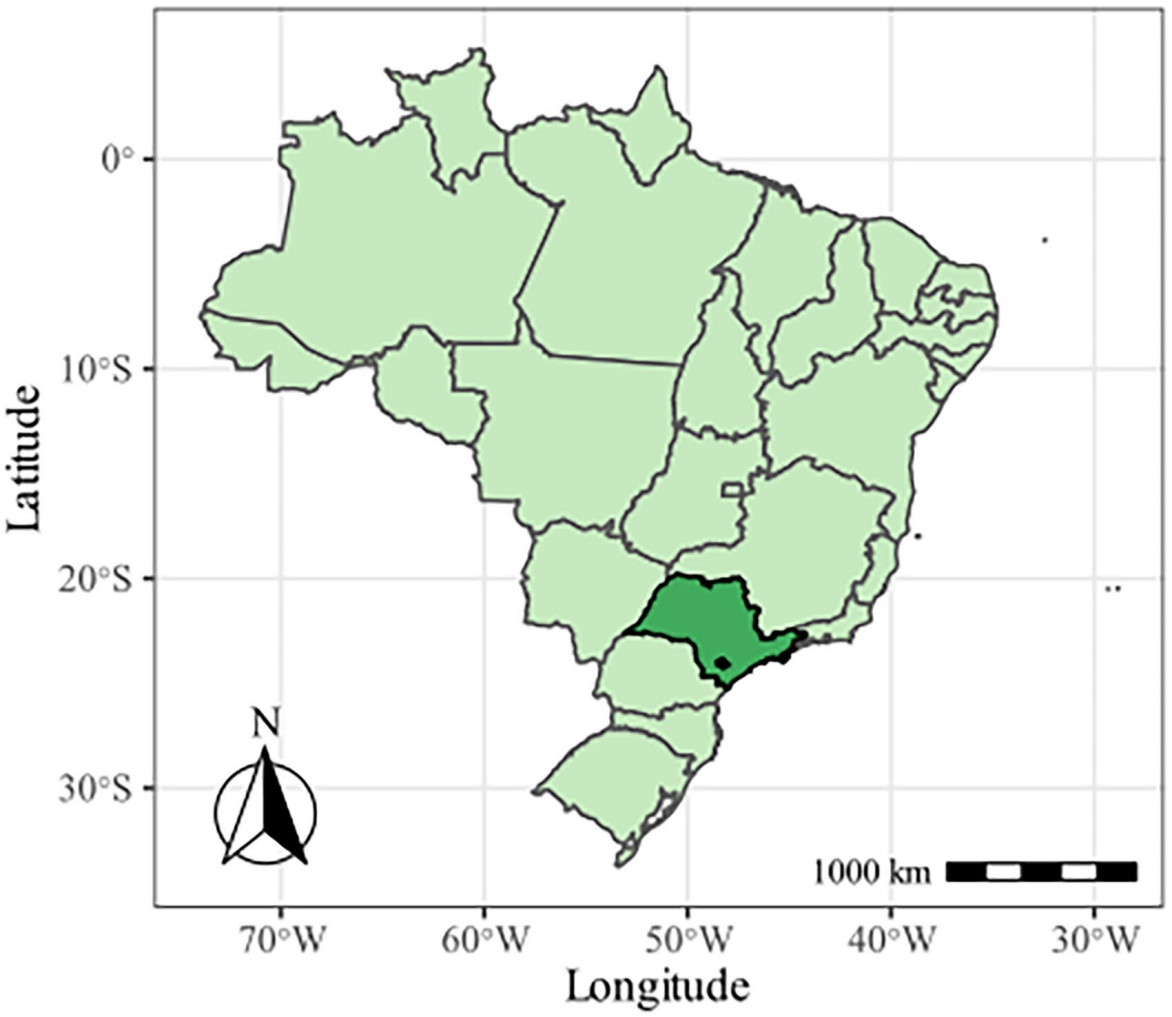
Location of the experimental area in Capão Bonito, SP, Brazil.

The experiment was carried out with soybean crop (*Glycine max* L. Merril) grown under commercial field conditions during the cropping seasons of 2018/2019 (application of treatments with Se) and 2019/2020 (assessment of residual effects of Se previously applied) at the Uva Farm, located in Capão Bonito, State of São Paulo (SP), Brazil, at the following geographic coordinates: Lat: −24.040934, Lon: −48.262421 (Figure 1). The weather of the region is characterized as humid subtropical (Cfa), with an average rainfall of 1,628 mm and an average annual temperature of 18.8°C (Alvares et al., 2013).

The soil of the experimental region—Oxisol—is classified as Typic Hapludox (Soil Survey Staff, United States Department of Agriculture Natural Resources Conservation Service, 2014) and the chemical and physical properties are as follows, according to the methodology suggested by Brazilian Agricultural Research Company (EMBRAPA) (1997) [pH (H_2_O) = 6.0; H + Al = 2.96; Al = 0.06; P (Mehlich-1) = 34.8 mg dm^−3^; K = 148 mg dm^−3^; S = 4.11 mg L^−1^; CEC = 9.83 cmol_c_ dm^−3^; Ca = 5.05 cmol_c_ dm^−3^; Mg = 1.44 cmol_c_ dm^−3^; P-rem = 28.10 mg L^−1^; organic matter = 2.69 dag dm^−3^; clay = 510 g kg^−1^; silt =110 g kg^−1^; and sand =380 g kg^−1^].

The experiment was arranged in a randomized block split-plot design, with four replicates. The biofortification of soybean was tested applying four different fertilizers: (i) Conventional monoammonium phosphate (C-MAP); (ii) Conventional monoammonium phosphate + Se (C-MAP + Se); (iii) Enhanced-efficiency monoammonium phosphate (E-MAP); and (iv) Enhanced-efficiency monoammonium phosphate + Se (E-MAP + Se). Monoammonium phosphate was coated with the humic and fulvic substances. The C-MAP + Se and E-MAP + Se fertilizers were prepared by spraying Se to the fertilizer granule. For this purpose, the fertilizers were coated after the granulation with 500 mg kg^−1^ of Se (from a solution of sodium selenate—Na_2_SeO_4_, Sigma-Aldrich, Saint Louis, MO, United States). Considering that 80 kg ha^−1^ of P_2_O_5_ were applied as MAP (~50% P_2_O_5_), the addition of Se-rich fertilizers (500 mg Se kg^−1^) added a Se rate of 80 g ha^−1^.

The aforementioned fertilizers were applied to four soybean genotypes, as follows: M5917 (maturity group = 5.9), 58I60 LANÇA (maturity group = 5.8), TMG7061 (maturity group = 6.1), and NA5909 (maturity group = 6.2; all of them presenting indeterminate growth type). Thus, the experiment had a total of 16 treatments, with four replicates, totaling 64 experimental plots. The fertilizers comprised the plots and the split-plots were represented by the genotypes. Each experimental split-plot was 30 m long by 3 m wide (soybean row spacing at 0.5 m, totaling 90 m^2^). Planting was made with 14 seeds per meter and fertilization was carried out during the sowing at the soybean seeds line (localized placement) by applying 16 kg ha^−1^ of N, 80 kg ha^−1^ of P_2_O_5_, and 28 kg ha^−1^ of K_2_O.

After the soybean harvest (described next), wheat was sown in the area but was not harvested for analysis. After wheat, soybean was sown in the succeeding summer crop to evaluate the residual effect of Se associated with the previously soil-applied phosphate fertilizer. Selenium treatments were not applied in this second season with all following the standard management carried out at the Uva farm.

### Analysis of oxidative stress and antioxidant enzymes

The uppermost fully developed leaf (trifoliolate) from 10 plants during the first cropping season (2018/2019) were collected at the full pod stage (R4) to evaluate antioxidant enzymes and oxidative stress. The collected leaves were frozen immediately in liquid nitrogen and stored in a deep freezer at −80°C for subsequent analysis. After that, the frozen plant material (0.2 g) was macerated in a porcelain mortar with liquid nitrogen and polyvinylpolypyrrolidone (PVPP) and mixed with 1.5 ml of buffer solution (100 mM potassium phosphate at pH 7.8, 0.1 mM EDTA, and 10 mM ascorbic acid).

The extract was centrifuged at 13,000 *g* for 10 min at 4°C. The supernatant was collected for measuring the activities of the enzymes, as follows: superoxide dismutase (SOD; Giannopolitis and Ries, 1977), ascorbate peroxidase (APX; Nakano and Asada, 1981), and catalase (CAT; Havir and McHale, 1987). In addition to that, 0.3 g of macerated frozen material were homogenized with 1.5 ml of 0.1% trichloroacetic acid (TCA) and centrifuged at 12,000 *g* for 15 min at 4°C for hydrogen peroxide (H_2_O_2_; Velikova et al., 2000) and peroxidation lipid (MDA; Buege and Aust, 1978).

### Soil Se content

For the determination of total Se content (partially available) in the soil, one composite soil sample (coming from five subsamples distributed around the experimental plot) was collected in each experimental plot at the full pod stage (R4). The samples were dried, homogenized, ground with a mortar and agate pestle, and passed through a 100-mesh nylon sieve. A sample mass of 0.5 g was mixed with 5 ml of *aqua regia* (a mixture of HNO_3_ 65% and HCl 37%—1:3 v/v). The mixture/suspension was left to stand for 1 h, and the Teflon® vessels were hermetically sealed and heated in a Mars-5 microwave digestion oven (CEM Corp, Matthews, NC, United States) with a temperature set at 175°C and a controlled pressure of 0.76 MPa for 25 min. Next, the vessels were cooled to room temperature and the volume was completed to 40 ml with bidistilled water.

Selenium in the soil samples was analyzed by Graphite Furnace Atomic Absorption Spectrometry with Zeeman background correction and EDL lamp for Se (GFAAS; AAnalyst™ 800 AAS, Perkin Elmer). The calibration curve for Se measurements was obtained from a standard solution with 1 g L^−1^ of Se (≥98% of purity, Fluka, Buchs, Switzerland). The reference material used for soil Se concentration was SRM 2709a [San Joaquin Soil, from the National Institute of Standards & Technology (Gaithersburg, MD, United States)], which contains 1.5 mg kg^−1^ of Se. The mean recovery of Se in this certified material was 88%.

### Harvest and yield determination

After R8 stage, when 95% of pods have attained maturity and have a variety-dependent color of brown or tan (134 and 495 days after the treatment application for the first and second season, respectively), grains from the useful area of the experimental plot were harvested and weighed to determine crop yield. Grain moisture was measured using a portable meter (model G650i, Gehaka®) and grain yield was corrected to 13%. A sample of each harvested plot was ground in a Willey mill for the determination of Se, N, protein, and total free amino acids.

### Nitrogen and selenium content in grains

Nitrogen quantification was performed by the Kjeldahl method described by Bremner (1996). The extraction for determination of Se was obtained by acid digestion of 0.5 g of ground grain, in a microwave oven, following the USEPA 3051A method (Usepa, 2007). Selenium contents were performed using an inductively coupled plasma mass spectrometer (ICP-MS; PerkinElmer, model NexIon 2000 B, Waltham, United States).

To ensure the quality of the digestion process, a reference standard from the Institute for Reference and Measurement Materials (White Clover – BCR 402, IRMM, Geel, Belgium, with 6.70 mg Se kg^−1^) and a blank sample were added to each digestion batch. The detection limit (LOD) was obtained using Se measurement in seven blank extracts and was calculated from the Equation 1:


(1) LOD=(x+t×s)×d

where:

*x* = mean content of the substance of interest in seven blank samples.

*t* = Student value to 0.01 of probability.

*s* = standard deviation.

*d* = dilution.

The fraction of the applied Se that was incorporated in soybean grains (Se recovery) was calculated using the Equation 2 described below:


(2) $$\mathrm{Se}\;\mathrm{recovery}\;\left(\%\right)=\frac{\left(\mathrm{Se}\;\mathrm{treatment}-\mathrm{Se}\;\mathrm{control}\right)}{\mathrm{Se}\;\mathrm{rate}\;}\times 100$$

where:

Se recovery (%) = use efficiency of the Se rates applied in the soil by soybean grains (Se utilization percentage);

Se treatment (g ha^−1^) = Se contents in soybean grains from soybean plants grown in treatments that received Se applications, considering the yield obtained in each treatment;

Se control (g ha^−1^) = Se contents in soybean grains from soybean plants grown in treatments without Se applications, considering the yield obtained in each treatment; and

Se rate (g ha^−1^) = Se rates applied in the soil.

### Total free amino acids and protein

Total free amino acids were determined using the ninhydrin method (Yemm et al., 1954). The quantification of protein in the grains was determined by multiplying the value of the N content by 6.25.

### Statistical analysis

The obtained data were primarily tested for their normality (Shapiro–Wilk’s test) and homogeneity of variance (Bartlett’s Test). Then, they were submitted to ANOVA, and when significant, mean values of variables found for each treatment were compared by the Tukey test at 5%. Principal component analysis (PCA) was performed for the dataset of conventional or enhanced fertilizer. The Pearson’s linear correlation matrix (*p* < 0.05) was also carried out, aiming to validate clusters and potential relationships of Se application in soil and plant attributes as outcomes of PCA. The analyses were made using the R software (R Core Team, 2020).

## Results

### Soybean yield (cropping seasons of 2018/2019 and 2019/2020)

**Figure 2:**
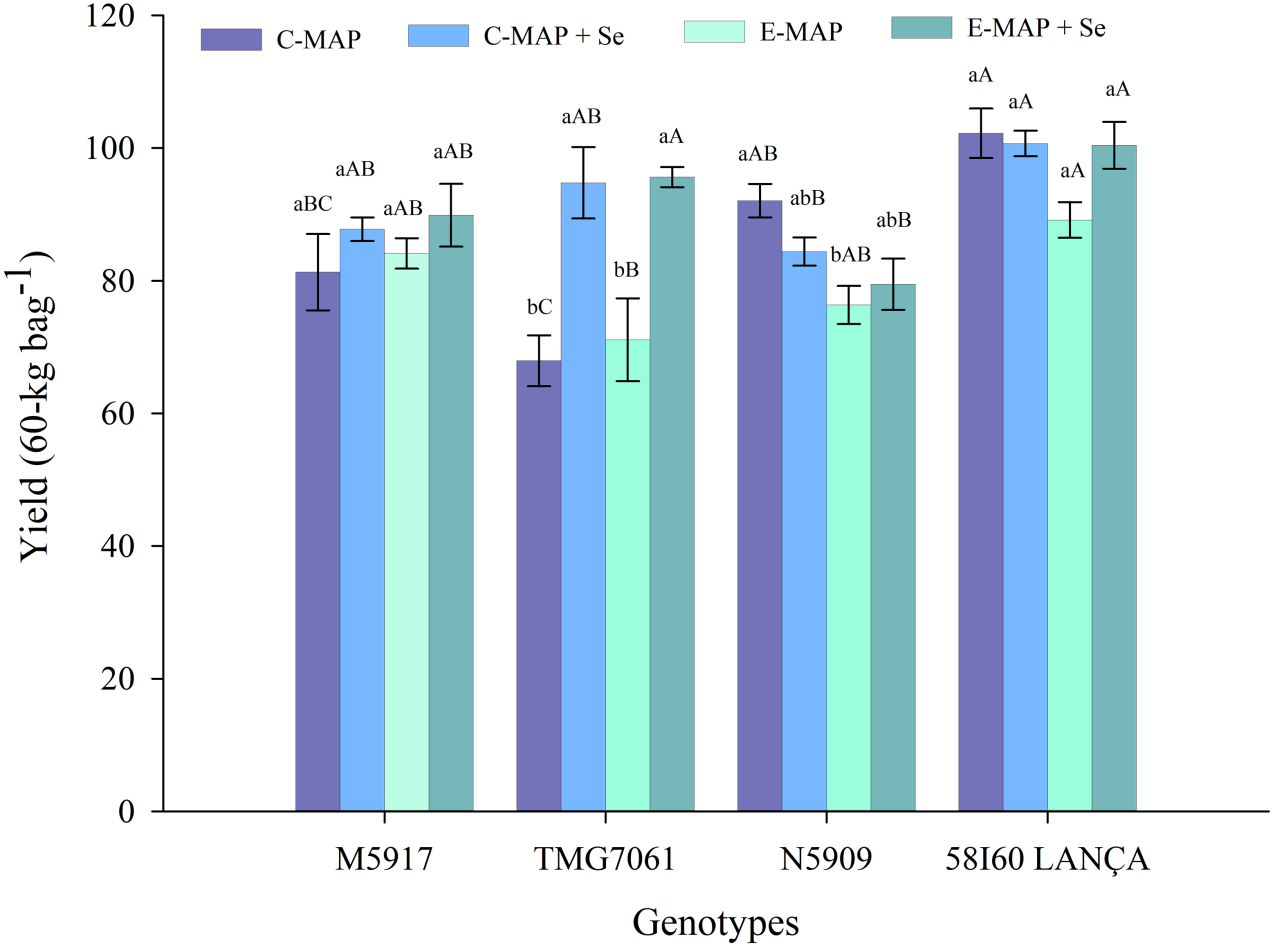
Yield (60-kg bags^−1^) of soybean grains harvested from the 2018/2019 cropping season. Lowercase letters compare soybean yields among fertilizers in each genotype and capital letters compare soybean yields among genotypes in each fertilizer source at the level of 5% (*p* < 0.05) by the Tukey test. The vertical bars refer to the standard error (*n* = 4). C-MAP, conventional monoammonium phosphate; C-MAP + Se, conventional monoammonium phosphate + Se; E-MAP, enhanced-efficiency monoammonium phosphate; and E-MAP + Se, enhanced-efficiency monoammonium phosphate + Se.

The tested factors (genotypes and fertilizer sources) affected soybean grain yield in the 2018/2019 season (*p* < 0.05). The fertilizer sources applied did not alter the yield of 58I60 LANÇA and M5917 genotypes. On the contrary, the genotype N5909 showed a statistical difference in yield by the Tukey’s test (*p <* 0.05), between the application of C-MAP and E-MAP, with 92.08 and 76.36 bags ha^−1^, respectively (Figure 2).

Grain yield in the TMG7061 genotype was higher in treatments using C-MAP + Se and E-MAP + Se when compared to C-MAP and E-MAP, reaching yields of 94.77 and 95.62 bags ha^−1^, respectively and gains of 24.51 and 26.85 bags ha^−1^ in yield, respectively. In the 2019/2020 cropping season, when the residual effect of Se applied in the soil was evaluated, the factors tested did not affect grain yield (*p* > 0.05; Supplementary Table 1).

### Selenium content in soybean grains and soil

**Figure 3:**
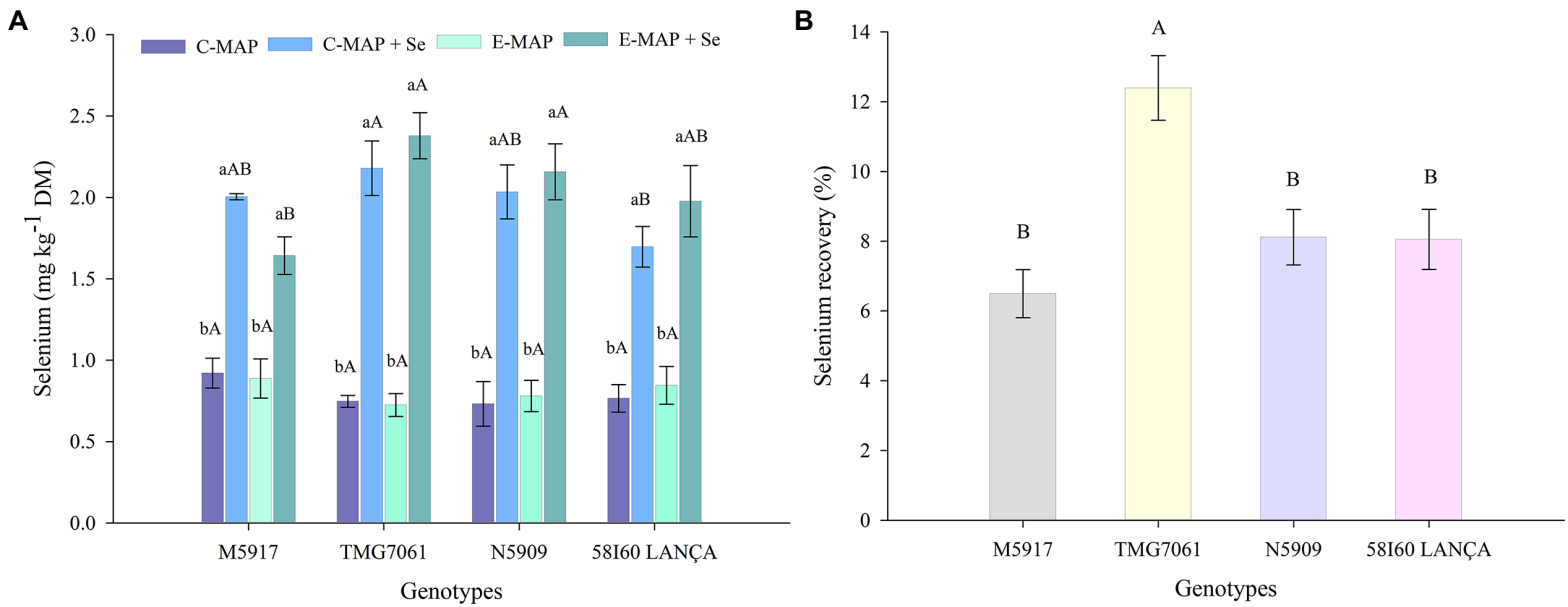
Selenium content (mg kg^−1^) and Se recovery (%) **(B)** in soybean grains harvested from the 2018/2019 cropping season. Lowercase letters compare Se contents and Se recovery among fertilizers in each genotype and capital letters compare Se contents and Se recovery among genotypes in each fertilizer source at the level of 5% (*p* < 0.05) by the Tukey test. The vertical bars refer to the standard error (*n* = 4). C-MAP, conventional monoammonium phosphate; C-MAP + Se, conventional monoammonium phosphate + Se; E-MAP, enhanced-efficiency monoammonium phosphate; and E-MAP + Se, enhanced-efficiency monoammonium phosphate + Se. The vertical bars refer to the standard error (**A**—*n* = 4; **B**—*n* = 8).

Selenium content analyzed in the reference material was 7.37 mg kg^−1^, indicating a recovery of 110%. The Se content in soybean harvested in the first season was influenced by the genotypes and sources of fertilizers applied (*p* < 0.05; Figure 3A). In all tested genotypes, the application of C-MAP + Se and E-MAP + Se increased the Se content in grains. In the genotype TMG7061, the increase in Se content was 2.90 and 3.31 times greater with the application of C-MAP + Se and E-MAP + Se, compared with their respective fertilizers without Se. In the other genotypes, the application of C-MAP + Se and E-MAP + Se presented values higher than two times the Se content accumulated into grains when the fertilizers C-MAP and E-MAP were applied.

Observing the content of Se in grains, with the use of C-MAP + Se and E-MAP + Se, the genotype TMG7061 presented the highest content, being however statistically different only from the genotype 58I60 LANÇA for C-MAP + Se and from the genotype M5917 for E-MAP + Se. The Se recovery by soybean grains was different among the tested genotypes (*p* < 0.05; Figure 3B), with the genotype TMG7061 showing the highest value (close to 12.4%).

The Se content in soybean grains harvested in the 2019/2020 crop was not influenced by the variables analyzed (*p >* 0.05; Supplementary Table 1). The average grain contents as a function of the fertilizers applied were 0.48 mg kg^−1^ (E-MAP), 0.52 mg kg^−1^ (C-MAP), 0.55 mg kg^−1^ (E-MAP + Se), and 0.62 mg kg^−1^ (C-MAP + Se). In the soil, the Se content did not differ statistically among treatments. The overall average Se content found in the soil in phase R4 was 0.73 mg dm^−3^, which justifies the low Se concentration in soybean grains of the crop carried out in the 2019/2020 cropping season (Supplementary Table 2).

### Nitrogen, protein, and amino acids

**Figure 4:**
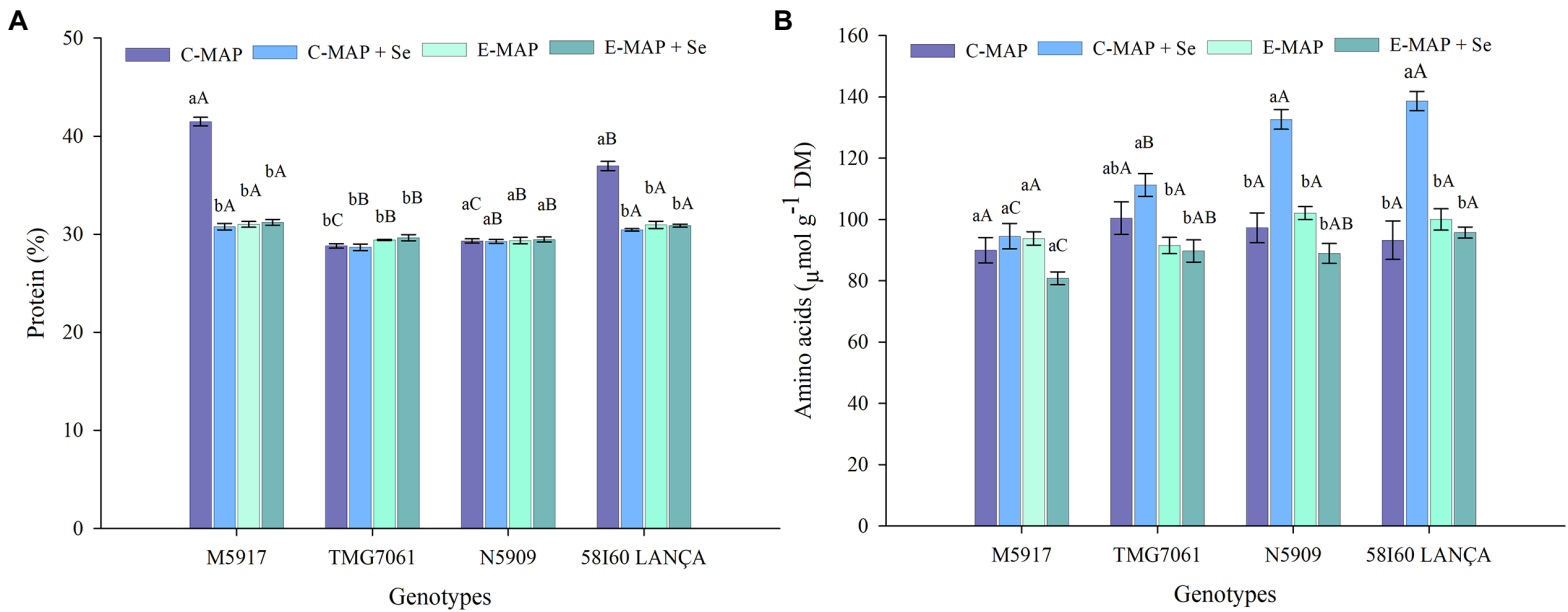
Protein (%) **(A)** and amino acids (μmol g^−1^ DM) **(B)** in soybean grains harvested from 2018/2019 cropping season. Lowercase letters compare protein and amino acids among fertilizers in each genotype and capital letters compare protein and amino acids among genotypes in each fertilizer source at the level of 5% (*p* < 0.05) by the Tukey test. The vertical bars refer to the standard error (*n* = 4). C-MAP, conventional monoammonium phosphate; C-MAP + Se, conventional monoammonium phosphate + Se; E-MAP, enhanced-efficiency monoammonium phosphate; and E-MAP + Se, enhanced-efficiency monoammonium phosphate + Se.

Nitrogen content, proteins, and total free amino acids were affected by the interaction between the tested genotypes and fertilizers. Following the application of C-MAP, the genotypes M5917 and 58I60 LANÇA showed higher N and protein contents compared with other treatments (Figure 4A; Supplementary Table 1). The total free amino acid content was higher with C-MAP + Se than with the other fertilizer sources for genotypes N5909 and 58I60 LANÇA (Figure 4B). Total free amino acid contents did not change due to the fertilizer sources applied for genotype M5917, whereas for genotype TMG7061, the highest and lowest values were verified after the application of C-MAP + Se and E-MAP and E-MAP + Se, respectively.

### Antioxidative metabolism

Overall, the activity of enzymes was not affected by the different fertilizers sources (Table 1). Superoxide dismutase and CAT had different activities among the genotypes, while APX was not affected by any of the factors under study. The genotype TMG7061 showed lower SOD activity and lower H_2_O_2_ concentration. Among the sources of fertilizers applied, C-MAP presented higher H_2_O_2_ content (2.09 μmol H_2_O_2_ g^−1^ MF), yet it differed only from the treatment with the application of E-MAP + Se (1.54 μmol H_2_O_2_ g^−1^ MF).

**Table 1:** Effect of Se application *via* soil on the activities of superoxide dismutase (SOD), catalase (CAT), ascorbate peroxidase (APX), lipid peroxidation by the MDA, and hydrogen peroxide (H_2_O_2_) with SEs (*n* = 4).

Genotype	Fertilizer	SOD (U SOD min^−1^ g^−1^ FM)	CAT (ηmol H_2_O_2_ min^−1^ g^−1^ FM)	APX (ηmol ASA min^−1^ g^−1^ FM)	MDA (ηmol MDA g^−1^ FM)	H_2_O_2_ (μmol H_2_O_2_ g^−1^ FM)
M5917	C-MAP	610.82 ± 18.66	2.97 ± 0.16	26.38 ± 2.01	15.94 ± 1.94 aAB	2.16 ± 0.23
TMG7061		616.14 ± 15.12	3.78 ± 0.47	29.37 ± 1.90	19.84 ± 0.58 aA	1.73 ± 0.27
N5909		647.83 ± 12.53	2.99 ± 0.30	23.55 ± 2.40	17.48 ± 1.45 aA	2.35 ± 0.09
58I60 LANÇA		658.31 ± 19.96	2.71 ± 0.29	23.36 ± 1.71	12.65 ± 1.31 aB	2.13 ± 0.24
M5917	C-MAP + Se	618.59 ± 31.55	3.36 ± 0.57	24.79 ± 2.29	13.40 ± 0.86 aA	2.00 ± 0.22
TMG7061		532.09 ± 25.74	3.37 ± 0.62	23.82 ± 2.49	13.48 ± 1.15 bA	1.35 ± 0.27
N5909		637.13 ± 13.51	2.23 ± 0.20	21.09 ± 2.50	13.08 ± 1.13 aA	1.87 ± 0.32
58I60 LANÇA		642.33 ± 24.15	1.59 ± 0.18	23.20 ± 2.87	13.94 ± 0.84 aA	1.89 ± 0.12
M5917	E-MAP	611.05 ± 24.87	2.46 ± 0.67	22.93 ± 4.04	11.77 ± 0.69 aA	2.10 ± 0.35
TMG7061		536.86 ± 21.09	3.35 ± 0.63	22.09 ± 4.12	12.45 ± 0.30 bA	1.13 ± 0.23
N5909		583.80 ± 21.11	2.58 ± 0.70	21.93 ± 2.08	15.71 ± 2.27 aA	1.88 ± 0.23
58I60 LANÇA		613.77 ± 26.17	2.69 ± 0.68	23.71 ± 3.31	14.25 ± 1.46 aA	2.11 ± 0.21
M5917	E-MAP + Se	619.08 ± 26.87	3.56 ± 0.47	28.84 ± 5.57	11.50 ± 1.42 aB	1.53 ± 0.20
TMG7061		546.71 ± 14.76	2.60 ± 0.57	21.29 ± 4.71	14.23 ± 0.59 bAB	0.97 ± 0.13
N5909		600.64 ± 18.22	2.78 ± 0.30	27.38 ± 4.84	12.86 ± 1.72 aAB	1.53 ± 0.29
58I60 LANÇA		639.01 ± 13.65	2.66 ± 0.77	26.23 ± 3.41	16.34 ± 1.93 aA	2.17 ± 0.28
M5917	General average to genotypes	614.88 ± 11.63 A	3.09 ± 0.25 AB	25.73 ± 1.77 ns	13.16 ± 0.75 ns	1.95 ± 0.13 A
TMG7061		557.95 ± 12.44 B	3.27 ± 0.28 A	24.14 ± 1.34 ns	15.00 ± 0.81 ns	1.29 ± 0.13 B
N5909		617.35 ± 10.06 A	2.65 ± 0.20 AB	23.49 ± 1.54 ns	14.78 ± 0.91 ns	1.91 ± 0.13 A
58I60 LANÇA		638.36 ± 10.47 A	1.09 ± 0.27 B	24.13 ± 1.34 ns	14.30 ± 0.73 ns	2.08 ± 0.10 A
C-MAP	General average to fertilizers	633.27 ± 16.57 ns	3.11 ± 0.30 ns	25.66 ± 2.00 ns	16.48 ± 1.32 ns	2.09 ± 0.21 a
C-MAP + Se		607.54 ± 23.74 ns	2.64 ± 0.39 ns	23.23 ± 2.54 ns	13.47 ± 0.99 ns	1.78 ± 0.23 ab
E-MAP		586.37 ± 23.31 ns	2.77 ± 0.67 ns	22.67 ± 3.39 ns	13.55 ± 1.18 ns	1.81 ± 0.26 ab
E-MAP + Se		601.36 ± 18.37 ns	2.90 ± 0.53 ns	25.93 ± 4.63 ns	13.73 ± 1.42 ns	1.55 ± 0.22 b

Lowercase letters compare among fertilizers in each genotype and capital letters compare among genotypes in each fertilizer source at the level of 5% (*p* < 0.05) by the Tukey test. No significance analysis was performed for SOD, CAT, APX, and H_2_O_2_ within soybean genotypes, as this was not the purpose of this study. C-MAP, conventional monoammonium phosphate; C-MAP + Se, conventional monoammonium phosphate + Se; E-MAP, enhanced-efficiency monoammonium phosphate; and E-MAP + Se: enhanced-efficiency monoammonium phosphate + Se. ns, no significant.

Malonaldehyde (MDA) levels were affected by the interaction between genotypes and fertilizers (*p* < 0.05), with genotype TMG7061 being the only one that showed a difference among fertilizers. In this genotype, MDA levels were higher with the application of C-MAP, indicating an increase in lipid peroxidation.

### Principal component analysis

**Figure 5:**
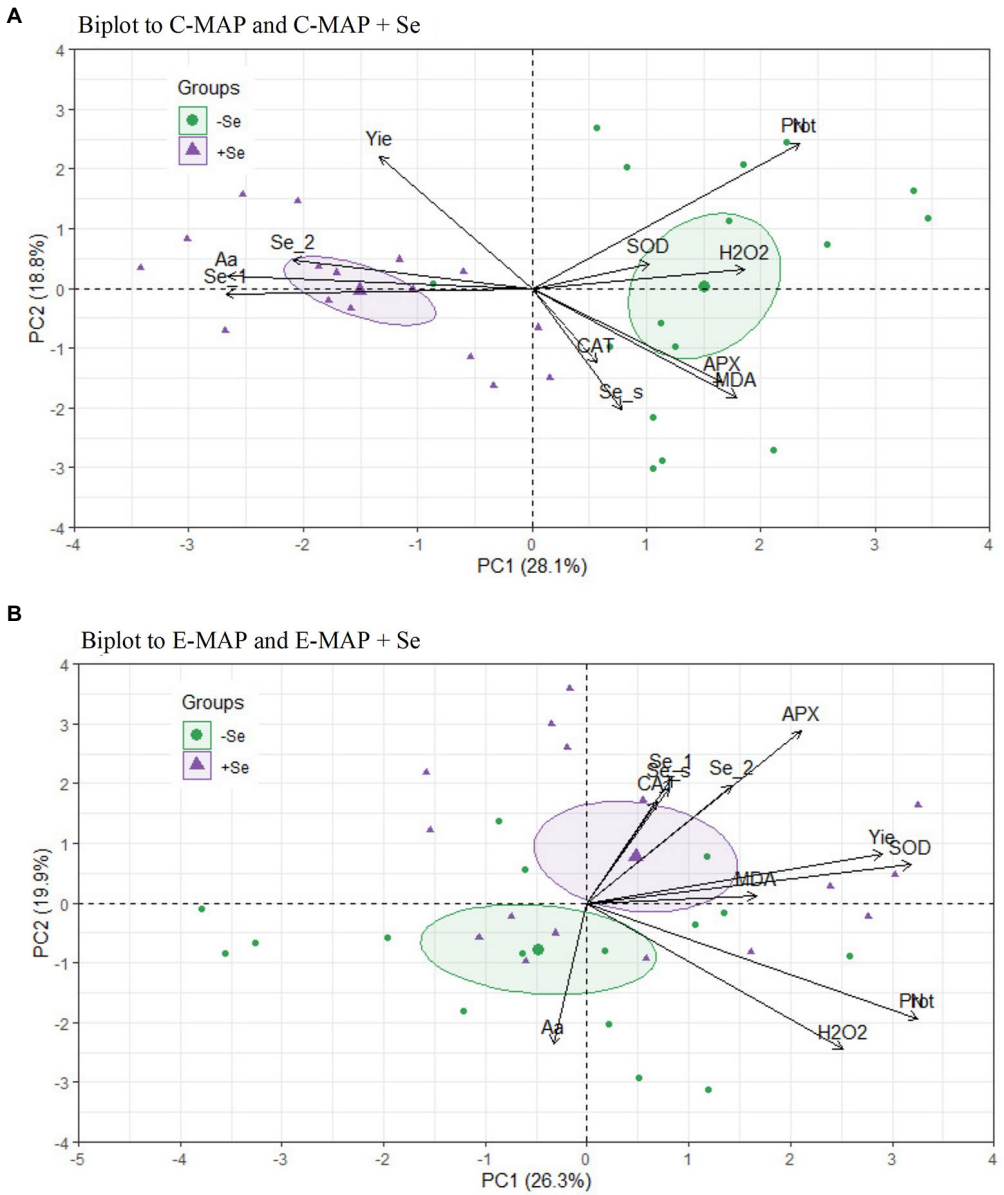
Biplot of principal component analysis (PCA) separated according to the fertilizers, **(A)** C-MAP and C-MAP + Se and **(B)** E-MAP and E-MAP + Se. Se content in grains of the cropping season of 2018/2019 (Se_1), Se content in grain of the cropping season of 2019/2020 (Se_2), Se in soil (Se_s), yield (Yie), protein in grains (Prot), amino acids in grains (Aa), lipid peroxidation (MDA), hydrogen peroxide (H_2_O_2_), catalase (CAT), superoxide dismutase (SOD), and ascorbate peroxidase (APX).

With the application of the conventional MAP with and without Se (C-MAP and C-MAP + Se), 46.9% of the covariances were explained by the PC1 and PC2 axes (Figure 5A). For E-MAP and E-MAP + Se, 46.2% of the covariances were explained by the PC1 and PC2, but the confidence intervals overlapped (Figure 5B). For fertilizers C-MAP and C-MAP + Se, the PCA showed that the concentration of total free amino acids correlates positively with the application of Se. In addition, the soybean grain yield from the cropping season of 2018/2019 was favored by Se application. The significance of the correlation among the studied variables was confirmed by Pearson’s linear correlation matrix (*p* < 0.05; Supplementary Figure 1).

## Discussion

### Yield

The average yield found in this study (89.7 bags ha^−1^) was above the national average (50.0 bags ha^−1^; Conab, 2022). This high average yield is related to the management adopted by the Uva farm and to the high soil fertility, based on soil attributes and nutrient concentration (e.g., P and K). To establish homogeneity in the final stand of plants and because all field operations were performed using commercial planting machines, the number of seeds that were sown per linear meter was the same for all genotypes, even though a higher number of seeds per linear meter was recommended for genotype TMG7061. Due to the presence of a larger stand of plants for this genotype (TMG7061), lodging of the plants occurred during the grain filling stage. Under high planting density, the light capture is reduced, reducing photosynthetic activity and carbohydrate accumulation in the stem, which leads to lodging (Song et al., 2020).

In addition to the high average yield, Se application increased grain yield for the TMG7061 genotype (Figure 2). The response of Se application to plant yield may vary depending on the genotype used (Thavarajah et al., 2015; Liu et al., 2021; Sher et al., 2022). At present, there are still very few specific reports on Se application in the soybean yield. In the principal component analysis, this increase in yield, correlated better with Se in the grains of soybean, when the plant was grown in soil fertilized with C-MAP + Se fertilizer (Figure 5A). In the work carried out by Deng et al. (2021), soil Se application also increased soybean yield compared with a control treatment. Previous studies have shown that Se can improve growth and increase antioxidant capacity in plants, which can affect yield, mainly when plants are exposed to stress factors (Boldrin et al., 2013; Nawaz et al., 2015; Mateus et al., 2021; Ravello et al., 2021).

### Enzymes

It has previously been established that Se can mitigate oxidative stress due to ROS regulation. This regulation can occur by stimulating the dismutation of O^2-^ into H_2_O_2_, by the regulation of enzymatic and non-enzymatic compounds, by the direct elimination of ROS by Se species, and by regulation of photosynthetic compounds (Silva et al., 2020). With Se application *via* C-MAP + Se, the MDA production was negatively correlated with Se content in grains, i.e., the production of MDA by leaves was lower as the Se content in grains increased (Supplementary Figure 1A). This reduction in MDA production demonstrates a clear ability to control ROS and thus oxidative stress, maintaining the integrity of cell membranes, allowing the maintenance of photosynthetic and productive performance of the plant, in addition to increasing Se contents in grains.

The activity of SOD and CAT enzymes was not influenced by the Se application, yet the formation of hydrogen peroxide was higher with the application of C-MAP, compared with E-MAP + Se in all genotypes (Table 1). In addition, the genotype TMG7061 was more sensitive to this change than the others, resulting in higher production of MDA when C-MAP was applied. However, the higher production of hydrogen peroxide acted as a priming/beneficial stress effect, allowing the plant to adjust for grain yield, not exceeding its limit of physiological plasticity capacity, which could lead to a decrease in productivity (Agathokleous et al., 2020). According to the PCA and the Pearson’s correlation matrix, ascorbate peroxidase activity in plants treated with Se application *via* E-MAP is positively correlated with Se content in grains. Lessa et al. (2020) showed that CAT, SOD, and APX activity had minimal interference from Se application *via* soil or leaf in rice, at a dose of 80 g ha^−1^.

According to Djanaguiraman et al. (2005), Se foliar application to soybean (50 ppm) increased the activity of SOD, glutathione peroxidase (GSH-Px), and proline, causing a decrease in lipid peroxidation and the reduction in plant senescence. The activity of stress mitigation enzymes, such as SOD, is increased under conditions with high ROS production. Moreover, with adequate levels of Se, the enzyme GSH-Px acts on the spontaneous reduction of O˙^2-^ (Hartikainen et al., 2000; Feng et al., 2013).

### Nutritional quality of grains and Se content in the soil

The average Se content found in the studied soil (0.73 mg dm^−3^) is within the range of Se contents reported for soils of the State of São Paulo (where the Uva farm is located), which varies from <0.08–1.61 mg dm^−3^. Soil Se content is influenced by characteristics such as pH (Schiavon et al., 2020), presence of competing ions such as sulfate and phosphate (Lessa et al., 2016; Santos et al., 2022), soil texture (Araujo et al., 2018), organic matter (Li et al., 2017), and presence of microorganisms (Gregorio et al., 2006).

Selenium content in grains harvested in the first crop season was higher in all genotypes with Se application, either *via* C-MAP + Se or *via* E-MAP + Se (Figure 3A). Considering the daily soybean intake of 50 g per person and the concentration of 2.37 mg kg^−1^ of Se in grains with the application of E-MAP + Se in the N5909 genotype, the concentration of Se ingested would be 118.5 μg day^−1^, a value that lies above the average daily intake of Se recommended for adults (70 μg day^−1^; Kipp et al., 2015).

The consumption of soybean by humans, for the most part, occurs indirectly as in the case of soybean sauce. The production of soybean sauce using biofortified soybean with Se is an alternative to increasing the Se intake by population utilizing supplementation of dietary change. Indeed, soybean sauce represents a strong antioxidant system, which keeps Se stable and non-toxic during storage (Gao et al., 2019, 2022). A study carried out by Gao et al. (2022) showed that soybean sauce produced from soybeans containing 259 μg kg^−1^ of Se contains 79.2 μg kg^−1^ of Se, with 24.8% being inorganic Se and 75.2% existing as organic Se form. This suggests that it is possible to produce a biofortified sauce using Se-enriched soybeans in the field.

The Se recovery observed in soybean ranged from 6.49 to 9.74% (Figure 3B). These values were higher than those reported by Lessa et al. (2020), who worked with soil Se fertilization in rice (maximum recovery = 2.7) and by Lara et al. (2019), who studied foliar application of Se in wheat (maximum recovery = 3%). This higher Se recovery by soybean grains can be attributed to its high protein concentration (about 40%). In the plant, sulfur present in selected amino acids can be replaced by Se, forming selenoaminoacids, which later form selenoproteins (White, 2016). Chan et al. (2010) found that selenospecies - including SeCys and SeMet - represent about 74% of the Se total in soybean grains, when this crop was treated with sodium selenite. Again, such results reinforce that soybean is an effective species when considering the biofortification of crops with Se.

Another factor that may have contributed to the greater Se recovery in soybean is the Se application associated with phosphate fertilizer. According to Qingyun et al. (2016), soils with nutrient deficiencies, especially P, may lead to reduced accumulation of Se in grains by crops. Phosphorus in soils occurs in anionic forms, which means that Se (as selenite—NaSeO_4_^−^) might compete with phosphate molecules for adsorption sites. However, the rates of phosphate fertilizers are much higher (nearly three orders of magnitude) than the amount of Se applied in this trial, making the retention of P more likely in these soils instead of the retention of Se.

In tropical soils, this competition between phosphate and selenite as well as between selenate and sulfate due to chemical similarities between them is acknowledged in the literature (Lessa et al., 2016; Lopes et al., 2017). The selenate adsorption process occurs mainly *via* formation of outer-sphere complexes, i.e., thru non-specific adsorption. However, for selenite, the formation of inner-sphere complexes occurs with the exchange of ligands, as well as phosphate, which for the most part is irreversible (McBride, 1994).

In the 2019/2020 cropping season, Se content in grains was lower (0.54 mg kg^−1^) than the first season, and there was no difference among treatments (Supplementary Table 1). This shows that there is a low residual effect of the soil-applied Se in the 2018/2019 season, irrespectively of the fertilizer applied, mainly after the cultivation of a winter crop (wheat). The low residual effect can be confirmed by the low Se concentration found in the soil in the R4 development phase (soil sampling time) during the first crop season. Indeed, studies have reported that part of the soil-applied Se can be fixed within a few months after application, making it unavailable for plant uptake (Gissel-Nielsen and Bisbjerg, 1970; Mikkelsen et al., 1989), which might be especially relevant for the case of the oxidic soil used in this study.

When applied as selenate, Se is found to be more available in soils than selenite in the short term. However, over time, Se^VI^ can be reduced to lower valence state species (e.g., Se^IV^), leading to further adsorption of the reduced species onto surfaces, including Fe/Mn/Al oxides. This effect occurs faster in acidic soils than in alkaline soils (Wang et al., 2017). Indeed, Ramkissoon et al. (2021) have reported that when selenate was applied in an Oxisol (pH = 6.8 and clay = 52%), 75% was adsorbed during the first day, which impaired the quantification of soluble Se 300 days after the application. The authors presumed that the oxides present in soil were responsible for Se sorption in this case. By contrast, soluble Se have decreased only 29% on a calcareous soil (pH = 8.2 and clay = 13%) after 300 days (Ramkissoon et al., 2021). This fact supports our findings, indicating that the low residual effect of Se at the second season is most likely related to selenium adsorption by soil.

In soils with low Se concentration (e.g., tropical regions), Se supply *via* fertilization is essential for biofortification strategies, especially in areas with no or low Se addition. However, the beneficial effects of fertilizer Se carried out in one season does not persist and that successive applications, associated with the application of other oxyanions that can compete with Se for oxidic sorption sites (e.g., phosphate and sulfate) as well as the addition of organic compounds *via* soil tend to increase the residual effect of Se in the soil (Qingyun et al., 2016). Indeed, the application of NPK fertilizer, associated or not with organic compost, has been reported to increase Se availability by 38.39 and 33.04% over 20 years (Qingyun et al., 2016).

The amount of total protein in soybeans was not increased by Se treatment. This fact supports the findings made by Yang et al. (2003) and Deng et al. (2022). However, the application of C-MAP + Se increased the free total amino acid content in genotypes N5909, Lança, and TMG7061. The results were consistent with previous studies indicating that an increase of Se in the crop could promote amino acids synthesis and thus improve amino acid content of Se-enriched soybean grains (Zhao et al., 2019).

## Conclusion

This present study showed that the application of C-MAP + Se and E-MAP + Se fertilizers is a promising method for biofortifying soybean with Se in tropical soils. This fact was especially relevant in the TMG7061 genotype when, the application of these fertilizers increases crop yield. In addition, the TMG7061 genotype showed greater recovery of Se by the grains. In summary, soybean is a good crop to be used in biofortification programs due to its high protein content and high capacity of Se recovery by the grains. Lastly, it is noteworthy the positive effect of the application of C-MAP + Se in grain quality, as it not only increased Se but also the amino acids content in the grains.

## Data availability statement

The original contributions presented in the study are included in the article/Supplementary material, further inquiries can be directed to the corresponding author.

## Author contributions

MAS: conceptualization, resources, writing—original draft, and writing—review and editing. GFS: conceptualization, resources, and writing—review and editing. APBC, JLL, and GSD: resources and writing—review and editing. CO: writing—review and editing. GL, DA, and PB: conceptualization and writing— review and editing. LRGG: conceptualization, funding acquisition, resources, and writing—review and editing. All authors contributed to the article and approved the submitted version.

## Funding

This work was financially supported by funds from the National Council for Scientific and Technological Development (CNPq), the Coordination for the Improvement of Higher Education Personnel (CAPES), the Foundation for Research of the State of Minas Gerais (FAPEMIG), and ICL South American Group.

## Conflict of interest

GSD was employed by ICL South American.

The remaining authors declare that the research was conducted in the absence of any commercial or financial relationships that could be construed as a potential conflict of interest.

GSD declares that this study received support from ICL South American. The funder had the following involvement in the study: providing support for travel expenses to/from the field experiments and helping with sample collection.

## Publisher’s note

All claims expressed in this article are solely those of the authors and do not necessarily represent those of their affiliated organizations, or those of the publisher, the editors and the reviewers. Any product that may be evaluated in this article, or claim that may be made by its manufacturer, is not guaranteed or endorsed by the publisher.
