# Supplementary material for: Selenium biofortification of soybean genotypes in a tropical soil via Se-enriched phosphate fertilizers
Source: Front Plant Sci. 2022 Sep 14;13:988140. doi: 10.3389/fpls.2022.988140 (PMC9517938; doi:10.3389/fpls.2022.988140)
Supplement: Supplementary file 1 [file Data_Sheet_1.docx]

Supplementary Material

# Supplementary Figures and Tables

## Supplementary Tables

**Supplementary Table 1.** Analysis of variance (ANOVA).

| Variable | Fertilizer | Genotype | Fertilizer x Genotype |
| --- | --- | --- | --- |
| Yield 2018/2019 | * | *** | *** |
| Yield 2019/2020 | ns | ns | ns |
| Selenium 2018/2019 | *** | ns | ** |
| Selenium 2019/2020 | ns | ns | ns |
| Selenium recovery | ns | * | ns |
| Selenium in soil | ns | ns | ns |
| Amino acids | *** | *** | *** |
| Nitrogen | *** | *** | *** |
| Protein | *** | *** | *** |
| SOD | ns | *** | ns |
| CAT | ns | * | ns |
| APX | ns | ns | ns |
| MDA | ns | ns | ** |
| H_2_O_2_ | ** | *** | ns |

* Significance 0.05; ** Significance 0.01; *** Significance 0.001; ns = no significance by F test.

**Supplementary Table 2.** Selenium in soil, Se in grain (2019/2020 season), N content, and S content in grains.

| Genotype | Fertilizer | Selenium in soil (mg dm^-3^) | Selenium in grain - 2019/2020 (mg kg^-1^) | N (g kg^-1^) |
| --- | --- | --- | --- | --- |
| M5917 | C_MAP | 0.558 ± 0.28 | 0.445 ± 0.01 | 66.38 ± 0.70 |
| TMG7061 |  | 0.924 ± 0.10 | 0.473 ± 0.08 | 46.11 ± 0.37 |
| N5909 |  | 0.782 ± 0.24 | 0.624 ± 0.06 | 46.90 ± 0.34 |
| 58I60 LANÇA |  | 0.623 ± 0.11 | 0.563 ± 0.09 | 56.14 ± 0.74 |
| M5917 | C-MAP + Se | 0.950 ± 0.30 | 0.614 ± 0.06 | 49.23 ± 0.53 |
| TMG7061 |  | 0.705 ± 0.18 | 0.602 ± 0.06 | 45.88 ± 0.54 |
| N5909 |  | 0.724 ± 0.33 | 0.598 ± 0.07 | 46.83 ± 0.31 |
| 58I60 LANÇA |  | 0.488 ± 0.16 | 0.699 ± 0.04 | 48.70 ± 0.21 |
| M5917 | E-MAP | 0.911 ± 0.23 | 0.488 ± 0.09 | 49.61 ± 0.47 |
| TMG7061 |  | 0.765 ± 0.17 | 0.361 ± 0.04 | 47.08 ± 0.09 |
| N5909 |  | 0.614 ± 0.12 | 0.634 ± 0.13 | 46.97 ± 0.53 |
| 58I60 LANÇA |  | 0.656 ± 0.07 | 0.469 ± 0.07 | 49.54 ± 0.59 |
| M5917 | E-MAP + Se | 0.694 ± 0.19 | 0.634 ± 0.01 | 49.95 ± 0.47 |
| TMG7061 |  | 0.740 ± 0.23 | 0.556 ± 0.10 | 47.45 ± 0.49 |
| N5909 |  | 0.976 ± 0.27 | 0.556 ± 0.06 | 47.15 ± 0.39 |
| 58I60 LANÇA |  | 0.703 ± 0.13 | 0.477 ± 0.04 | 49.40 ± 0.24 |

## Supplementary Figures


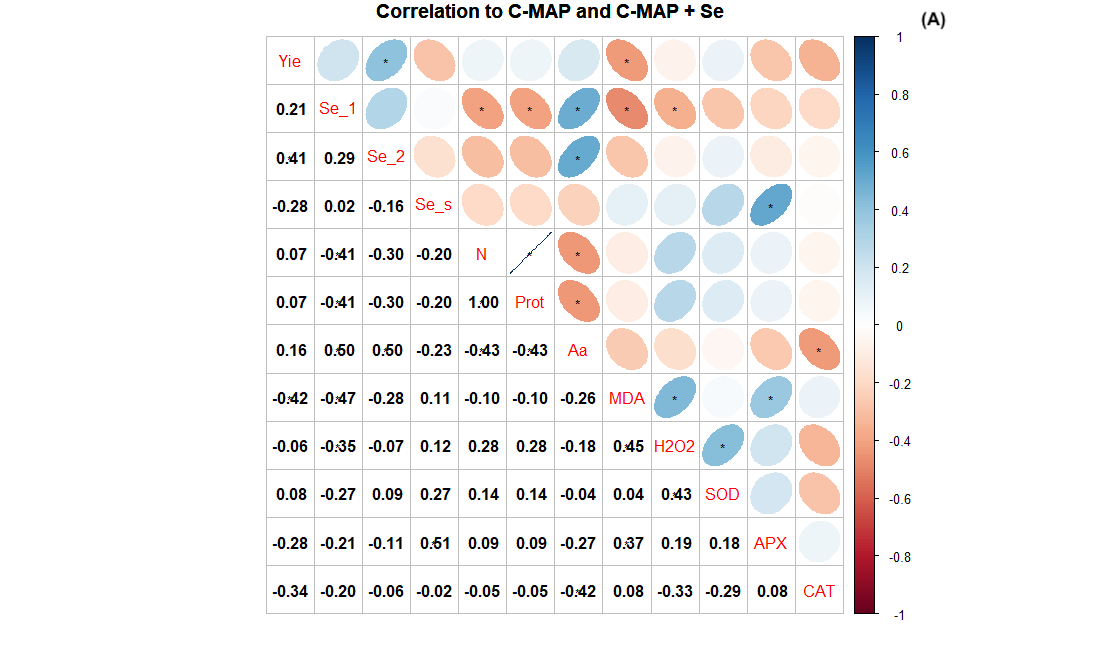

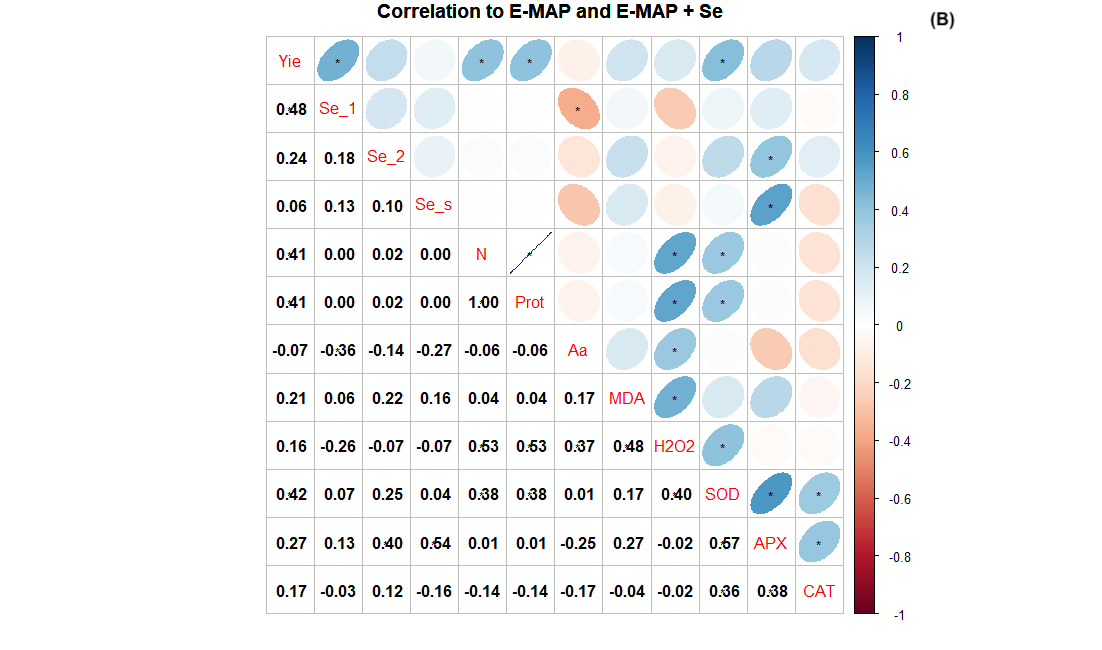


**Supplementary Figure 1.** Pearson’s linear correlation matrix to C-MAP and C-MAP + Se (A) and to E-MAP and E-MAP + Se (A). * significant relationship of soil and plant attributes at p < 0.05; blue ellipse with right sloping top: positive relationship; red ellipse with left sloping top: negative correlation. Se content in the grain 2018/2019 (Se_1), Se content in the grain 2019/2020 (Se_2), Se in soil (Se_s), yield (Yie) protein in grains (Prot), amino acids in grains (aa), lipid peroxidation (MDA), hydrogen peroxide (H_2_O_2_), catalase (CAT), superoxide dismutase (SOD), ascorbate peroxidase (APX).
